# Transcriptional Suppression of the NLRP3 Inflammasome and Cytokine Release in Primary Macrophages by Low-Dose Anthracyclines

**DOI:** 10.3390/cells9010079

**Published:** 2019-12-28

**Authors:** Nilay Köse-Vogel, Sven Stengel, Elena Gardey, Tatiana Kirchberger-Tolstik, Philipp A. Reuken, Andreas Stallmach, Tony Bruns

**Affiliations:** 1 Department of Internal Medicine IV (Gastroenterology, Hepatology, and Infectious Diseases), Jena University Hospital, Friedrich Schiller University of Jena, 07747 Jena, Germany; nilay.koese@med.uni-jena.de (N.K.-V.); sven.stengel@med.uni-jena.de (S.S.); elena.gardey@med.uni-jena.de (E.G.); tatiana.kirchberger-tolstik@med.uni-jena.de (T.K.-T.); philipp.reuken@med.uni-jena.de (P.A.R.); andreas.stallmach@med.uni-jena.de (A.S.); 2 The Center for Sepsis Control and Care (CSCC), Jena University Hospital, Friedrich Schiller University of Jena, 07747 Jena, Germany; 3 Department of Internal Medicine III, University Hospital RWTH Aachen, 52074 Aachen, Germany

**Keywords:** inflammation, macrophages, innate immunity, innate immune memory, histones, epigenetics

## Abstract

Tissue-resident macrophages play critical roles in controlling homeostasis, tissue repair, and immunity. Inflammatory macrophages can sustain tissue damage and promote the development of fibrosis during infections and sterile tissue injury. The NLRP3 inflammasome and its effector cytokine IL-1β have been identified as important mediators of fibrosis. Epirubicin, an anthracycline topoisomerase II inhibitor, has been reported to inhibit myeloid inflammatory cytokine production and to promote tissue tolerance following bacterial infection. We investigated the anti-inflammatory properties of epirubicin on the NLRP3 inflammasome and TLR4-mediated inflammation in PMA-primed THP-1 and in primary human peritoneal macrophages (PM). Low-dose epirubicin at non-cytotoxic doses downregulated NLRP3 inflammasome components and reduced the release of cleaved caspase-1, bioactive IL-1β, and TNF-α following NLRP3 activation in a dose-dependent fashion. In addition, epirubicin attenuated inflammatory macrophage responses after TLR4 and TLR2 ligation. These anti-inflammatory effects were not mediated by the induction of autophagy or altered MAPK signaling, but as the result of a global transcriptional suppression of LPS-dependent genes. Epirubicin-treated macrophages displayed reduced acetylation of histone 3 lysine 9 (H3K9ac), suggesting anti-inflammatory epigenetic imprinting as one underlying mechanism.

## 1. Introduction

Inflammation is a host response towards destructive stimuli that aims at limiting tissue damage and restoring homeostasis [1]. The inflammatory host response needs to be tightly regulated, as the unrestricted release of inflammatory mediators can lead to pathological fibrosis, impairing normal tissue function and promoting organ failure and death [2]. In addition to their role in promoting wound healing responses and tissue repair [3], organ-resident macrophages have been shown to contribute to the development of organ fibrosis by promoting tissue injury, maintaining pro-fibrotic responses, or blocking resolution pathways [4,5]. Accumulating evidence suggests a critical role of the myeloid inflammasomes as an important driver of organ fibrosis in various pathophysiological and clinical settings [6,7,8].

The inflammasomes are cytoplasmic multiprotein complexes, which mediate the cleavage of caspase-1, leading to the maturation and release of the pro-inflammatory cytokines interleukin (IL)-1β and IL-18 [9]. Among the NLR family, pyrin domain-containing 3 (NLRP3) is characterized best and can be activated by a variety of host- and pathogen-derived stimuli, such as bacterial pore-forming toxins, extracellular ATP, reactive oxygen species (ROS), crystals, and protein aggregates [9]. Recent studies have shown that the activation of the NLRP3 inflammasome in myeloid, epithelial, and mesenchymal cells promotes liver injury and fibrosis [8,10,11]. Although IL-1β is well known to drive pro-fibrotic responses in the liver, lung, and kidneys in experimental models [12,13,14], these data suggest a role of the inflammasome beyond the mere IL-1β release, also implying tumor necrosis factor (TNF) [8]. Therefore, therapeutic strategies that suppress the activation of the NLRP3 inflammasome and the release of IL-1β and TNF-α from inflammatory macrophages may represent a promising strategy to modulate inflammation, organ damage, and fibrosis.

Epirubicin is an anthracycline chemotherapeutic agent, which impedes topoisomerase II-mediated strand relegation, thereby causing double-stranded DNA breaks (DSB) leading to DNA damage and cell death [15]. In light of recent observations that epirubicin mitigates inflammatory cytokine release and organ damage in inflammatory models in vivo and in vitro [16], we investigated anti-inflammatory properties of epirubicin on the NLRP3 inflammasome and TLR4-mediated inflammation in PMA-primed THP-1 and human primary peritoneal macrophages (PM) as an approach to limit myeloid-driven inflammation and fibrosis.

## 2. Materials and Methods

### 2.1. Cell Line and Primary Cells

The human monocytic cell line THP-1 (ACC-16, DSMZ, Braunschweig, Germany) and primary human peritoneal macrophages (PM) were used. PM were isolated from ascitic fluid (AF) from patients with decompensated cirrhosis and ascites in the absence of peritoneal carcinomatosis, peritonitis, and acute pancreatitis. The study conformed to the ethical guidelines of the 1975 Declaration of Helsinki and was approved by the internal review board (Ethics committee of the Jena University Hospital, 3683-02/3). Patients granted written informed consent prior to inclusion.

Mononuclear cells were isolated by using density gradient separation with lympholyte-H (Cedarlane, Burlington, Canada). PM were purified from mononuclear cells by magnetic assisted cell sorting (MACS) using the anti-human CD14 antibody conjugated MicroBeads (Miltenyi Biotec, Bergisch Gladbach, Germany) according to manufacturer’s instructions. Flow cytometry revealed a purity of >95%.

### 2.2. Cell Cultures

Cells were cultivated in RPMI-1640 medium (Biochrom AG, Berlin, Germany) supplemented with 10% fetal bovine serum (FBS; Pan Biotech, Aidenbach, Germany) and 1% L-glutamine-penicillin-streptomycin solution (GPS; Sigma-Aldrich, St. Louis, Missouri, USA). Prior to further stimulation, THP-1 were differentiated with 100 ng/mL phorbol 12-myristate 13-acetate (PMA; Abcam, Cambridge, UK) for 18 h.

Cells were treated with freshly prepared, sterile-filtered epirubicin (Sigma Aldrich) at concentrations between 0.01 to 5 µg/mL for 24 h, unless stated otherwise. After epirubicin treatment, PM and THP-1 were stimulated for 3 h with LPS from *E*.*coli* K12 (InvivoGen, San Diego, CA, USA) at concentrations of 10 or 100 ng/mL, respectively, followed by treatment with freshly prepared 1 mM ATP (InvivoGen, San Diego, CA, USA) for 1 h. For the inhibition of autophagy, 300 nM bafilomycin (Invivogen, San Diego, CA, USA) or 10 mM 3-methyl adenine (3-MA; Invivogen, San Diego, CA, USA) were added to the cells simultaneously with LPS, and maintained until the end of the experiment. For TLR2 ligation, cells were incubated either with 10^8^ cells/mL of heat-killed listeria monocytogenes (HKLM; Invivogen, San Diego, CA, USA) or 100 ng/mL Pam3CSK4 (Invivogen, San Diego, CA, USA) for 4 h.

### 2.3. Enzyme-Linked Immunosorbent Assay (ELISA)

For detection of the released caspase-1 and cytokines IL-1β, TNF, and IL-1RA in cell culture supernatants, ELISA were performed according to the manufacturer’s instructions (caspase-1: R&D systems, Minneapolis, Minnesota, USA; IL-1β, TNF-α, and IL-1RA: Thermo Fisher Scientific, Waltham, MA, USA).

### 2.4. IL-1β Bioactivity Assay

The bioactivity of secreted IL-1β in the cell culture medium was investigated with HEK-Blue IL-1β reporter cell line (Invivogen, San Diego, CA, USA) and subsequent colorimetric assay with Quanti-Blue (Invivogen, San Diego, CA, USA), according to manufacturer’s instructions.

### 2.5. Cytotoxicity Assays

Cytotoxicity was determined by measuring the succinate dehydrogenase activity as a marker of metabolic activity of the cells using tetrazole MTT. THP-1 was incubated with 0.5 ng/mL MTT solution for 30 min, while PM was incubated with 0.5 ng/mL MTT solution for 2 h. MTT was replaced by solubilization buffer (90% isopropanol, 10% Triton-X, 1 drop 5 M HCl) and mixed by pipetting up and down. Absorbance was measured by a plate reader (Infinite M200 Pro, Tecan, Männedorf, Switzerland) at 570 nm. Metabolic activity in percentage (%) was calculated by setting the untreated control cells to 100%.

As lactate dehydrogenase (LDH) release into cell culture medium correlates with cell death, cytotoxicity was assessed by the LDH assay using the Pierce LDH cytotoxicity assay kit (Thermo Fisher Scientific, Waltham, MA, USA) according to the manufacturer’s instructions. Maximum LDH activity of cultured cells was determined by lysing the cells 30 min before the end of the experiment with lysis buffer supplied in the kit. To calculate the % cytotoxicity, LDH activity of untreated control was subtracted from the treated sample LDH activity, divided by the total LDH activity (which is determined by subtracting LDH activity of untreated control from maximum LDH activity), and multiplied by 100.

Early and late apoptosis were quantified using annexin V and 7-aminoactinomycin D (7-AAD) staining protocols according to the manufacturer’s instructions (BD Biosciences, New Jersey, USA) and analyzed by flow cytometry (CytoFlex, Beckman Coulter, Brea, CA, USA). Unstained cells, cells stained with annexin-V alone, and cells stained with 7-AAD alone were used to define cell populations and quadrants. Cells that were both annexin-V and 7-AAD negative were considered as alive, cells that were annexin-V positive and 7-AAD negative were defined as early apoptotic; whereas cells that were both annexin-V and 7-AAD positive were defined as late apoptotic.

### 2.6. FLICA Assay

In order to measure active caspase-1 and subsequent pyroptosis levels, fluorochrome-labeled inhibitors of caspases (FLICA) 660 Caspase-1 assay kit (Immunochemistry Technologies, Bloomington, MN, USA) was used according to company’s recommendations. Briefly, epirubicin- or LPS/ATP-treated cells were detached from the cell culture plate, washed, and incubated in 1:60 diluted FLICA solution for 1 h at 37 °C in 5% CO_2_. Following incubation, the cells were washed with washing buffer. Prior to flow cytometry analysis, 1 μL propidium iodide (PI) was added to each FACS tube and the cells were immediately analyzed by flow cytometry (CytoFlex, Beckman Coulter, USA).

### 2.7. Flow Cytometry

Cell surface staining of CD14 was performed using a FITC-labelled anti-human CD14 antibody (BD Biosciences, USA) clone M5E2 for 30 min at 4 °C in PBS containing 2% FCS and 2 mM EDTA. Intracellular staining was performed using the Cytofix/Cytoperm fixation/permeabilization kit (BD Biosciences, USA) and an APC-labelled TNF-alpha antibody (Biolegend, San Diego, CA, USA, Clone MAb11) or isotype-matched controls. Cells were stained with LIVE/DEAD™ fixable aqua dead cell stain kit (Thermo Fisher Scientific, USA) prior to permeabilization for intracellular staining. Prior to intracellular staining and 45 min after LPS treatment, cells were treated with 5 ng/mL brefeldin A until the end of the experiment. After staining and subsequent washing steps, cells were immediately analyzed by flow cytometry using adequate isotype-matched and fluorescence-minus-one controls (Cytoflex, Beckman Coulter, USA).

### 2.8. Protein Extraction, SDS-PAGE, and Western Blot

For whole cell lysates, cells were harvested in ice-cold radioimmunoprecipitation assay (RIPA) buffer (150 mM NaCl, 1.0% Nonidet P-40, 0.5% sodium deoxycholate, 0.1% SDS, 50 mM Tris, pH: 8.0, protease inhibitor cocktail). Lysates were mixed, incubated on ice for 30 min with occasional vortexing, and centrifuged at 15,000× *g* at 4 °C for 15 min. Supernatants were transferred into new tubes and stored at −20 °C. Nuclear proteins (soluble nuclear extracts) were obtained by using a subcellular protein fractionation kit for cultured cells according to manufacturer’s instructions (Thermo Scientific, USA).

Protein concentrations were determined with the Micro BCA protein assay kit (Thermo Fisher Scientific, USA). Ten µg of cellular protein was subjected to sodium dodecyl sulfate polyacrylamide gel electrophoresis (SDS-PAGE) and transferred to polyvinylidene fluoride (PVDF) membranes (Thermo Fisher Scientific, USA) by immunoblotting. Immunostaining was performed with primary antibodies against the protein of interest and corresponding horseradish peroxidase-conjugated anti-rabbit or anti-mouse IgG secondary antibodies (R&D systems, USA). Chemiluminescence was measured using immobilon western chemiluminescent HRP substrate (Merck, Darmstadt, Germany) on a G-BOX Chemi XX6 imaging device (Syngene, Bangalore, India). The following antibodies were used at 1:1000 dilutions: anti-LC3B (L7543, Sigma, USA), anti-IL-1β (#12703, Cell Signaling Technology, Danvers, MA, USA), anti-caspase-1 (NB100-56565, Novus Biologicals, Centennial, Colorado, USA), anti-NLRP3 (#15101, Cell Signaling Technology USA), anti-phospho-histone H2A.X (Ser139) (05-636-I, Merck, Germany), anti-histone H3 (#4499S, Cell Signaling Technology, USA), anti-acetyl histone H3 (Lys9) (ABE18, Milipore, Burlington, MA, USA), IkBA (#9242, Cell Signaling Technology), NF-kB P65 (D14E12, #8242, Cell Signaling Technology), phospho-NF-kB P65 (SER536) (93H1, #3033, Cell Signaling Technology), phospho-p38 MAPK (Thr180/Tyr182) (#9211, Cell Signaling Technology), phospho-p44/42 MAPK (Erk1/2) (Thr202/Tyr204) (#9101, Cell Signaling Technology), and anti-β-actin (sc-47778, Santa Cruz Biotechnology, Dallas, Texas, USA).

ImageJ 1.49v software [17,18] was used to perform quantification of western blot images. A profile plot for each lane was created based on the density of the exact bands. The area of the peak was selected and calculated. Arbitrary units were normalized on the expression of the housekeeping proteins, as indicated in the figure legends.

### 2.9. Immunocytochemistry

Cultured cells were grown on cover slips in 24-well plates, fixed with 4% paraformaldehyde (PFA) in PBS, permeabilized with PBS containing 0.5% Triton-X, and blocked with blocking solution (1.5% BSA, 0.4% Triton X-100 in PBS) at RT for 1 h. Then, cover slips were incubated with mixtures of primary antibodies (anti-phospho-histone H2A.X (Ser139), 05-636-I, Merck, Germany, 1:300; anti-53bp1, NB100-304, Novus Biologicals, USA, 1:1000) and diluted in blocking solution at RT for 1 h. Afterwards, Alexa Fluor^®^ 488-conjugated donkey anti-rabbit and Cy-3 conjugated donkey anti-mouse (Jackson Immunoresearch, Cambridgeshire, UK) solutions were applied at a dilution of 1:400. Finally, cover slips with stained cells were mounted with DAPI Fluoromount-G (Southern Biotech, Birmingham, AL, USA) on microscope slides and analyzed on an LSM 710 AxioObserver microscope (Zeiss, Jena, Germany).

### 2.10. Microarray Analysis

THP-1 treated with or without epirubicin (0.25 μg/mL) and with or without LPS were used in duplicates for microarray analysis performed by a commercial transcriptomics provider (Eurofins Genomics, Aarhus, Denmark) using the Affymetrix Clariom S HT 24-array plate according to standard procedures. Briefly, total RNA was isolated by using NucleoSpin^®^ RNA Plus kit (Macherey Nagel, Düren, Germany) according to the manufacturer’s instructions. The RNA concentration and purity were determined spectrophotometrically (DS-11 FX, DeNovix, Wilmington, Delaware, USA) and confirmed by RNA ScreenTape system (Tapestation 2200, Agilent, Santa Clara, CA, USA). Then, cDNA was synthesized from 100 ng RNA, in vitro transcribed, and labelled using a GeneChip WT Plus labeling kit. After labeling, the generated target RNA was loaded onto an array plate, stained, and imaged on the GeneTitan multichannel instrument (Thermo Fisher Scientific, Waltham, MA, USA. CEL files were load into a transcriptome analysis console (TAC) and signal space transformation robust multiarray average algorithm (SST-RMA) was applied for background correction, normalization, and probe set summarization.

### 2.11. RNA Extraction, cDNA Synthesis, and Quantitative Real-Time Polymerase Chain Reaction (qRT-PCR)

Total RNA was isolated from cultured and treated cells by using NucleoSpin^®^ RNA Plus kit (Macherey Nagel, Germany) according to manufacturer´s instructions. The RNA concentration and purity were determined spectrophotometrically (DS-11 FX, DeNovix, USA). Next, cDNA was synthesized by using the High-Capacity cDNA reverse transcription kit (Applied Biosystems, Foster City, CA, USA) according to the instructions of the manufacturer. Prior to qRT-PCR, cDNA was diluted 1:10 in nuclease-free water. qRT-PCR was performed with the gene-specific primers (Supplementary Table S1) using Maxima SYBR Green qPCR Master Mix (2X) (Thermo Fisher Scientific, USA) according to the recommended instructions of the manufacturer. Using Rotor-Gene Q (Qiagen, Hilden, Germany), the qRT-PCR reaction was performed with a two-step cycling program, with 1 cycle of 95 °C for 10 min for initial denaturation, followed by 40 cycles of 95 °C for 15 s and 60 °C for 1 min to detect and record the SYBR Green fluorescence during the annealing step of each cycle. Melting curves were generated for quality control. The 2^−ΔΔCt^ method was used to calculate the relative fold change [19].

### 2.12. Statistical Analysis

The statistical analysis was performed using GraphPad Prism 7 software (San Diego, CA, USA). Data are presented as mean ± standard error of the mean (SEM), unless stated otherwise. One- or two-way analysis of variance (ANOVA) with post-hoc test were performed for group comparisons. One sample t-test was used to compare the differences in normalized data. To test for significant correlation, the Spearman’s rank correlation coefficient was used.

For microarray analysis, differentially expressed genes were determined using empirical Bayes statistics for differential expression within TAC software. The enrichment analysis of differentially expressed genes was performed using Gene Set Enrichment Analysis (GSEA) tool from Broad Institute [20] using the following settings: permutation type—gene set; number of permutations—1000; gene set database—H: Hallmark gene sets were from Molecular Signature Database v6.2. Heatmaps, dot plots, and principal component analysis were conducted in R [21]. The statistical test applied for *p*-value calculation is noted in the legends of the respective figures. Results with *p* ≤ 0.05 were considered as statistically significant. Levels of significance were set as follows: *** *p* < 0.001; ** *p* < 0.01, * *p* < 0.05.

### 2.13. Data Accessibility

RNA microarray data from this study have been deposited in GEO with the accession code GSE136758 (https://www.ncbi.nlm.nih.gov/geo/).

## 3. Results

### 3.1. Priming with Low-Dose Epirubicin Suppresses NLRP3 Inflammasome Activity in Macrophages

**Figure 1:**
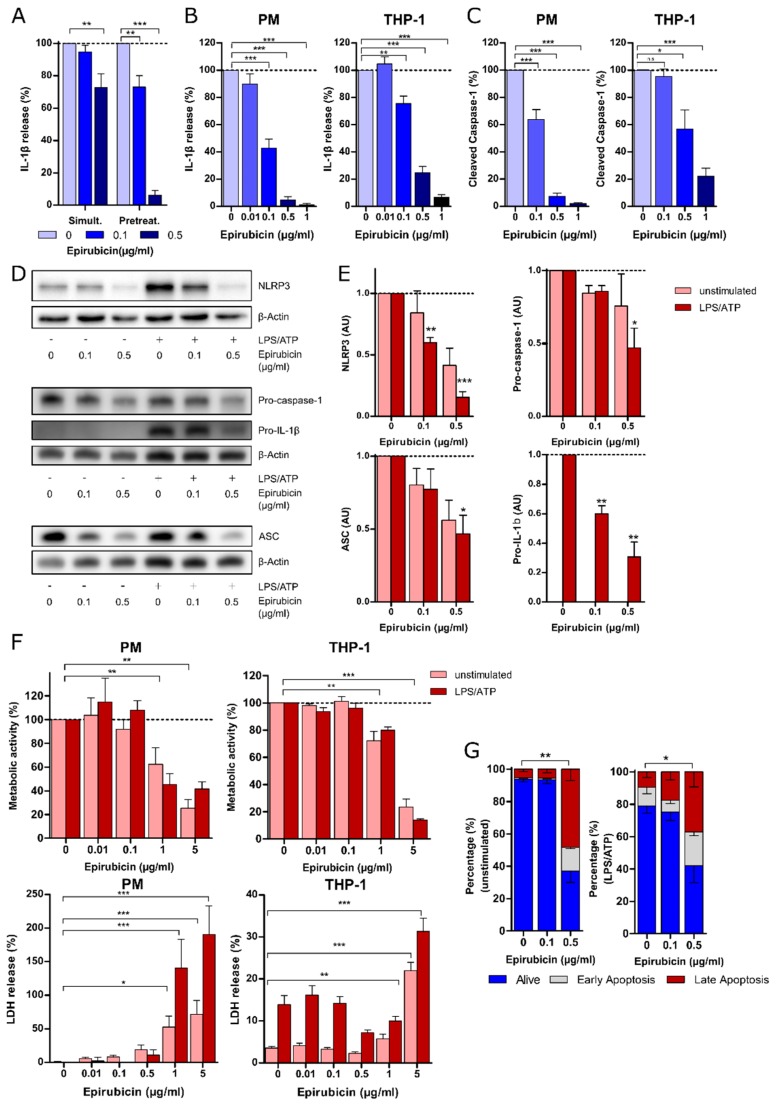
Epirubicin suppresses NLRP3 inflammasome activity. (**A**) PM were treated with epirubicin 24 h prior (Pretreat.) or simultaneously (Simult.) with LPS (10 ng/mL, 3 h) and subsequently with ATP (1 mM, 1 h). Cell culture supernatants were analyzed by ELISA for IL-1β (*n* = 4). (**B**,**C**) PMA-primed THP-1 or PM were incubated with increasing doses of epirubicin for 24 h and subsequently stimulated with LPS (10 or 100 ng/mL)/ATP. Supernatants were analyzed by ELISA for IL-1β (**B**) (*n* = 11 for PM, *n* = 6 for THP-1) and cleaved caspase-1 (**C**) (*n* = 6 for PM, *n* = 5 for THP-1). (**D**) Representative immunoblots of inflammasome components in the whole cell lysates of PM after epirubicin (24 h) and subsequent LPS/ATP. Here, β-actin is shown as loading control. (**E**) Quantitative analysis of NLRP3, pro-IL-1β, procaspase-1, and ASC protein bands by densitometry using Image J software and normalized to the density of β-actin. AU: Arbitrary units (*n* = 5). (**F**) Succinate dehydrogenase activity (metabolic activity) and LDH release (cytotoxicity) are shown in PM and THP-1 following epirubicin incubation in the absence or presence of inflammasome activation by LPS/ATP. Mean and SEM from 4–6 independent experiments are shown. (**G**) To assess early and late apoptosis, PM were stained with annexin-V and 7-AAD after treatment with epirubicin (24 h) in the absence or presence of inflammasome activation by LPS/ATP (*n* = 4). Percentage of alive cells was used for the statistical analysis. Statistical analysis: two-way analysis of variance (ANOVA) with Bonferroni test (**A**), one-way ANOVA with Tukey’s multiple comparison test (**B**,**C**,**G**), one-way ANOVA with Tukey’s multiple comparison test (F-LDH), one-sample *t*-test (**E**), one-sample *t*-test (F-MTT); n.s.: not significant; * *p* < 0.05, ** *p* < 0.01, *** *p* < 0.001.

In order to examine the effect of epirubicin on NLRP3 inflammasome activity in macrophages, we utilized an established in vitro model of NLRP3 inflammasome activation, in which extracellular ATP drives the oligomerization of NLRP3 and the auto-cleavage of caspase-1 in LPS-primed macrophages [9,22]. IL-1β release was reduced by prophylactic treatment with low-dose epirubicin at 0.1 µg/mL when administered 24 h before stimulation, but not when administered at the same time as LPS (Figure 1A). Epirubicin concentrations of 0.5 µg/mL or higher suppressed the IL-1β release from PM when administered simultaneously with LPS. In dose-finding studies, treatment with epirubicin in concentrations starting from 0.1 µg/mL was effective in reducing the release of IL-1β from PM and THP-1 (Figure 1B) and the release of cleaved caspase-1 from PM into cell culture supernatants (Figure 1C). Using an IL-1β reporter cell assay, we confirmed that epirubicin suppressed the release of bioactive IL-1β, and not the inactive pro-IL-1β (Supplementary Figure S1). Low-dose epirubicin significantly reduced NLRP3 and pro-IL-1β protein expression, whereas effects on ASC and pro-caspase-1 expression were less pronounced at 0.1 µg/mL (Figure 1D,E).

To rule out relevant cytotoxicity of epirubicin at this concentration, cell viability was determined using LDH and MTT assays, as well as annexin-V/7-AAD costaining. Using these methods, significant epirubicin-mediated cell death was detected at concentrations starting from 1 µg/mL epirubicin (Figure 1F). However, investigating apoptosis induction by costaining with annexin-V and 7-AAD clearly revealed that the induction of programmed cell death already started at concentrations of 0.5 µg/mL, whereas it was not found at concentration of 0.1 µg/mL (Figure 1G). Pyroptosis, as assessed by FLICA assay, was not affected by epirubicin (Supplementary Figure S2). Weighing anti-inflammatory effects against cell death, subsequent experiments focused on effective doses of 0.1 µg/mL (PM) and 0.1–0.25 µg/mL (THP-1) epirubicin.

### 3.2. No Evidence for Significant Double-Strand Break Damage Repair after Low-Dose Epirubicin

**Figure 2:**
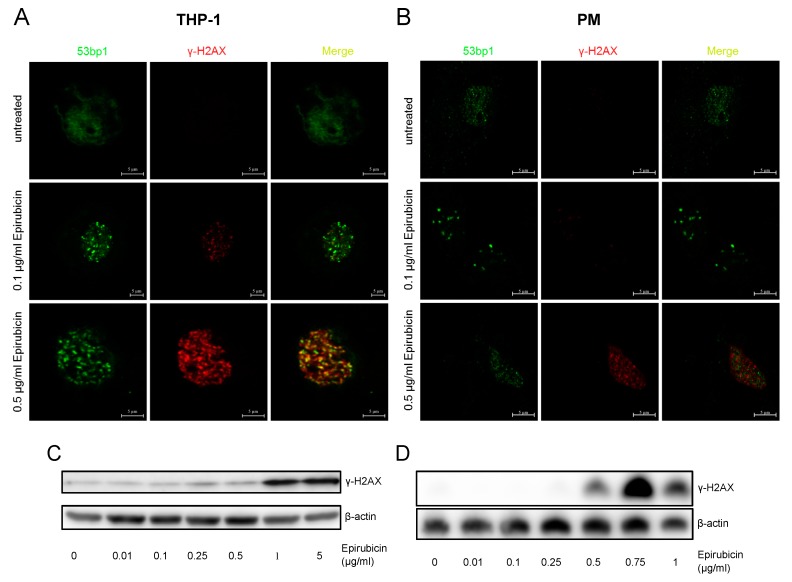
DNA damage repair in response to epirubicin. Representative confocal images and western blots of PMA-primed THP-1 and PM. (**A**) THP-1 and (**B**) PM were treated with indicated concentrations of epirubicin, and fixed and stained for 53bp1 (green) and γ-H2aX (red). Costaining is indicated by yellow color in merged images. Images were taken with a confocal laser scanning microscope with 63× objective (LSM 710, Zeiss) (Scale bars: 5 µm). Whole cell lysates of (**C**) THP-1 and (**D**) PM were subjected to SDS-PAGE and processed for immunoblotting analysis with γ-H2AX antibody. Here, β-actin served as a loading control.

The anti-tumor effect of high-dose epirubicin in cancer cells was found to be dependent on the induction of double-strand breaks, eventually leading to cell death [15]. The induction of foci of double-strand break (DSB) repair at anti-inflammatory, low-dose concentrations of epirubicin was investigated using confocal microscopy. THP-1 and PM treated with epirubicin did not exhibit increased colocalization of 53bp1 and γH2AX in immunofluorescence at 0.1 µg/mL, but did at higher concentrations (Figure 2A,B). Western blots confirmed increased γH2AX induction at concentrations ≥ 1 µg/mL in THP-1 and at ≥ 0.5 µg/mL in PM (Figure 2C,D), indicating that at effective anti-inflammatory concentrations epirubicin does not induce significant DNA damage responses.

### 3.3. The Anti-Inflammatory Effect of Low-Dose Epirubicin is Independent of Autophagy

**Figure 3:**
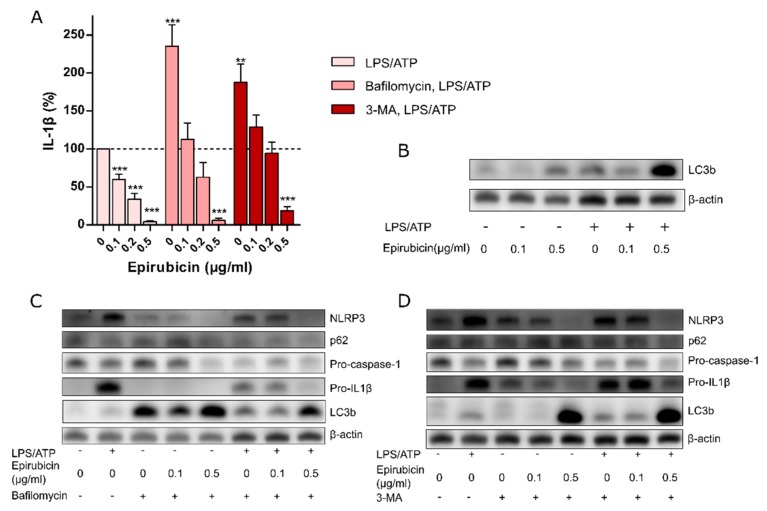
Epirubicin suppresses the NLRP3 inflammasome in presence of autophagy inhibitors. (**A**) LPS/ATP-induced IL-1β secretion from PM in the absence or presence of bafilomycin and 3-MA is shown. Means ± SEM from 10 (IL-1β) independent experiments are shown. (**B**) Representative immunoblot of LC3b after epirubicin and LPS/ATP treatments. (**C**,**D**) Representative immunoblots of inflammasome and autophagy components after inhibition with (**C**) bafilomycin or (**D**) 3-MA in PM whole cell lysates. Cells were treated with indicated concentrations of epirubicin treatment over 24 h, followed by LPS (10 ng/mL) in the absence or presence of bafilomycin (300 nM) or 3-MA (10 mM) for 3 h and NLRP3 activation by ATP (1 mM, 1 h). Whole cell lysates were subjected to SDS-PAGE (10% gel) and processed for immunoblot analysis with specific antibodies. Here, β-actin served as loading control. Statistical tests: one-sample *t*-test (**A**) ** *p* < 0.01, *** *p* < 0.001.

Previous studies demonstrated epirubicin as an enhancer of autophagy [23]. To investigate the role of autophagy in the suppression of inflammasome activity by low-dose epirubicin, 3-methyl adenine (3-MA) and bafilomycin were used to inhibit early and late autophagy, respectively [24,25]. As expected, the inhibition of autophagy increased IL-1β secretion from PM in response to LPS/ATP as compared to untreated controls (Figure 3A). However, the dose-dependent effect of epirubicin on IL-1β secretion prevailed in the presence of 3-MA or bafilomycin (Figure 3A). At non-cytotoxic doses (0.1 µg/mL), epirubicin did not change LC3b protein levels as a model substrate to measure autophagic flux. Only treatment with a higher dose of epirubicin (0.5 µg/mL), which we have shown to induce apoptosis, was accompanied by an accumulation of LC3b, indicating an impact on the autophagic flux (Figure 3B). In addition, the presence of autophagy inhibitors did not alter the epirubicin-mediated reduction of inflammasome components NLRP3, pro-caspase-1, or pro-IL1β at the protein level (Figure 3C,D), and it did not affect pyroptosis (Supplementary Figure S3). In the presence of bafilomycin or 3-MA, 0.5 µg/mL epirubicin resulted in an accumulation of LC3b protein and a minor decrease in p62 (Figure 3C,D). Together, these data indicate increased autophagic fluxes at higher concentrations of epirubicin only [26].

### 3.4. Low-Dose Epirubicin Suppresses TNF-α Release in Response to TLR4 and TLR2 Ligation

**Figure 4:**
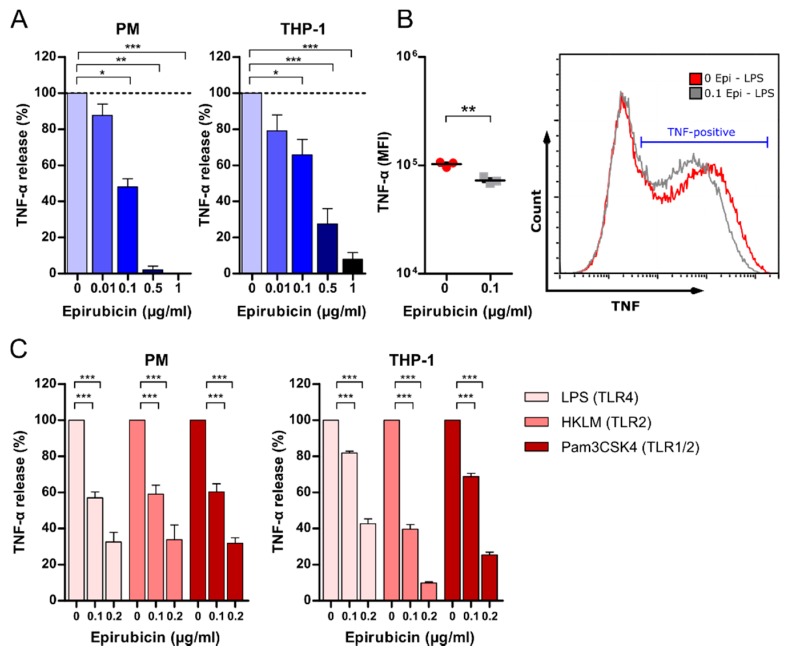
Epirubicin suppresses TNF-α release in response to TLR4 and TLR2 ligation. (**A**) PMA-primed THP-1 or PM was incubated with increasing doses of epirubicin for 24 h, and subsequently stimulated with LPS (10 or 100 ng/mL of PM or THP-1, respectively) for 3 h and ATP (1 mM) for 1 h. Cell culture supernatants were analyzed by ELISA for TNF-α (*n* = 7 for PM, *n* = 5 for THP-1). (**B**) Flow cytometry analysis showing intracellular TNF mean fluorescence intensity (MFI) in PMA-primed, LPS-stimulated THP-1. Epirubicin in µg/mL (Epi) (**C**) PM or PMA-primed THP-1 were incubated with epirubicin for 24 h, and subsequently treated with LPS (10 ng/mL), HKLM (10^8^ cells/mL), or Pam3CSK4 (100 ng/mL) for 4 h. Supernatants were analyzed by ELISA (*n* = 6 each). Statistical tests: one-way (**A**) or two-way (**B**) ANOVA with Bonferroni correction or unpaired *t*-test with Welch correction (**C**); * *p* < 0.05, ** *p* < 0.01, *** *p* < 0.001.

In addition to suppressing IL-1β, low-dose epirubicin also suppressed the release of TNF-α in PM and THP-1 in response to LPS/ATP in a dose-dependent manner (Figure 4A), albeit with high interindividual variability (Supplementary Figure S4). This was confirmed by flow cytometry using intracellular staining of TNF-α in LPS-stimulated PM (Figure 4B, Supplementary Figure S5). However, while the number of TNF-α producing cells did not change due to epirubicin treatment (Supplementary Figure S6), we observed reduction in intracellular TNF-α per cell as indicated by lower median fluorescence intensity (Figure 4B). Additionally, to assess the effects of low-dose epirubicin on the TNF-α release in response to TLR4 and TLR2 agonists, PM and PMA-primed THP-1 were treated with HKLM, Pam3CSK4, or LPS in the absence of ATP. TLR4- and TLR2-mediated TNF-α release was reduced after priming of THP-1 and PM with low-dose epirubicin (Figure 4C), indicating that low-dose epirubicin not only reduced NLRP3 inflammasome activity, but also attenuated macrophage inflammation in response to pathogen-associated molecular patterns (PAMP) other than LPS.

### 3.5. Low-Dose Epirubicin Suppresses the Global Transcriptional Expression of LPS-Dependent Genes

**Figure 5:**
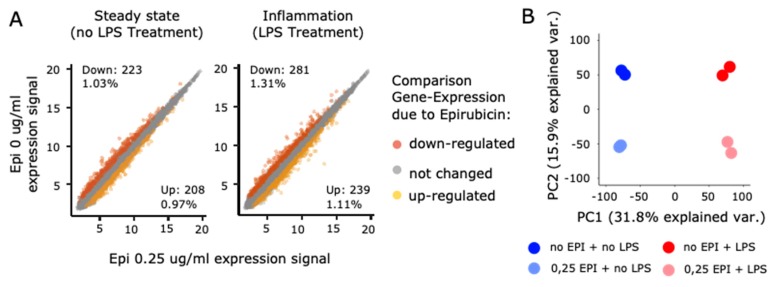

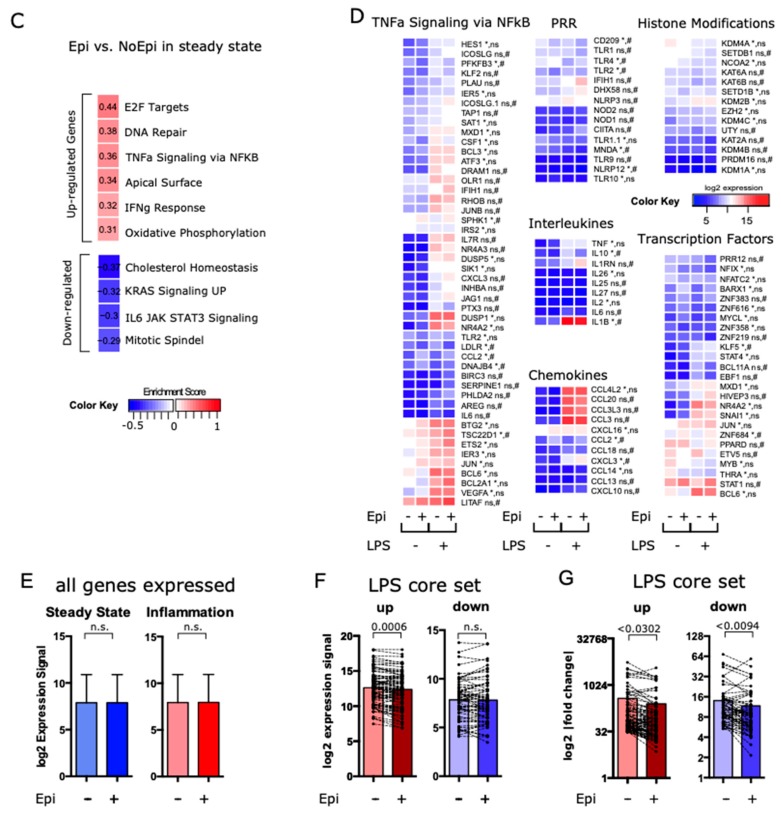
Epirubicin alters the gene expression patterns in THP-1. (**A**) Scatter plot of the expression signal of genes in the absence or presence of epirubicin. Upregulated genes (*p* < 0.01, fold change (FC) ≥ 2) are shown in yellow, while downregulated (*p* < 0.01, FC ≤ –2) genes are depicted in orange. (**B**) Unsupervised principle component analysis (PCA) showing the transcriptome differences between expression microarray data of epirubicin or LPS-treated THP-1. The percentages of the total variation that are accounted for by the 1st and 2nd principle components are shown. (**C**) Pathways with highest enrichment scores revealed by gene set enrichment analysis (GSEA) of genes in steady state conditions shown with enrichment score. (**D**) Representative heatmaps of genes regulated by epirubicin shown in their relation to pathways or gene families. Significant differences after epirubicin are indicated by * in absence of LPS and by # in presence of LPS. (**E**–**G**) Expression signals of (**E**) all expressed genes (mean and SD) and (**F**,**G**) genes of the LPS core set (FC ≥ 30 or FC ≤ −5, FDR ≤ 0.0001) in absence or presence of epirubicin priming and treatment with LPS are shown. Statistical test (**E**–**G**) Paired *t*-test, n.s. not significant.

To further characterize the suppressive effects of epirubicin on cellular inflammation, we performed genome-wide transcriptome analysis in the absence or presence of epirubicin (24 h) or LPS (3 h) (Supplementary Table S2). For these gene expression studies, we used the slightly higher but non-cytotoxic concentration of 0.25 µg/mL epirubicin in order to identify a more characteristic gene expression profile than estimated at 0.1 µg/mL in PMA-differentiated THP-1. In the absence of LPS, 430 genes (~2% of all genes analyzed) were found to be differentially regulated by epirubicin (*p*-value ≤ 0.01, fold change (FC) ≤ −2 or ≥2) (Figure 5A, left panel). After LPS, the number of genes regulated by epirubicin increased to 520 (~2.4% of all genes analyzed) (Figure 5A, right panel). Unsupervised principle component analysis (PCA) indicated discrete and stable clusters of cell transcriptomes undergoing different treatment conditions (Figure 5B).

Gene set enrichment analysis (GSEA) of epirubicin-dependent upregulated genes under unstimulated conditions revealed an enrichment of genes belonging to the E2F signaling pathway, DNA repair, and TNF signaling via NFκB, whereas downregulated genes were associated with IL6-JAK-STAT3 and others. Under inflammatory conditions, epirubicin treatment led to changes in inflammatory pathways, such as IFNa and IFNg signaling, as well as IL6-JAK-STAT3 signaling-dependent genes (Figure 5C, Supplementary Table S3). Low-dose epirubicin treatment effected NFκB-dependent TNF signaling and the regulation of *TLR1*, *TLR2*, *NLRP3*, and *CD209* (Figure 5D). Furthermore, *IL1B*, *IL6*, and *IL10* were downregulated following epirubicin treatment, whereas the interleukin-1 receptor antagonist encoding gene (*IL1RN*) was upregulated. In addition, genes coding for histone-modifying enzymes, including *KDM4A*, *KAT2A*, and *KAT6A*, were found to be differentially expressed after epirubicin treatment.

As epirubicin intercalates into DNA and inhibits transcription factor binding, resulting in attenuated regulation of RNA transcription [27], we hypothesized that the anti-inflammatory effect of epirubicin is due to reduced transcription regulatory capability. To test this hypothesis, we first analyzed the global effect on transcription by comparing the expression values of all expressed array genes in the absence or presence of epirubicin. We could not observe a significant shift in global gene expression (Figure 5E). Next, we investigated whether LPS-dependent transcription initiation might be impaired by epirubicin. To investigate this hypothesis, we determined an LPS gene core set for THP-1 by extracting highly regulated genes after LPS treatment from epirubicin that had not been treated with THP-1. In doing so, 88 upregulated genes (FC ≥ 30, FDR ≤ 0.0001) and 63 downregulated genes (FC ≤ −5, FDR ≤ 0.0001) were identified (Supplementary Table S4). Within the LPS core set, strongly upregulated genes exhibited significantly lowered expression values when cells were pretreated with epirubicin, whereas for genes downregulated by LPS, no statistically significant trend towards a reduced regulation was found (Figure 5F). After adjusting our analysis for gene expression changes induced by epirubicin (by calculating the relative FC induced by LPS), we could confirm that epirubicin significantly reduced both the induction as well as the reduction of LPS core set genes (Figure 5G).

### 3.6. Low-Dose Epirubicin Effects the Expression of Genes Involed in TLR4 and IL-1 Signaling

**Figure 6:**
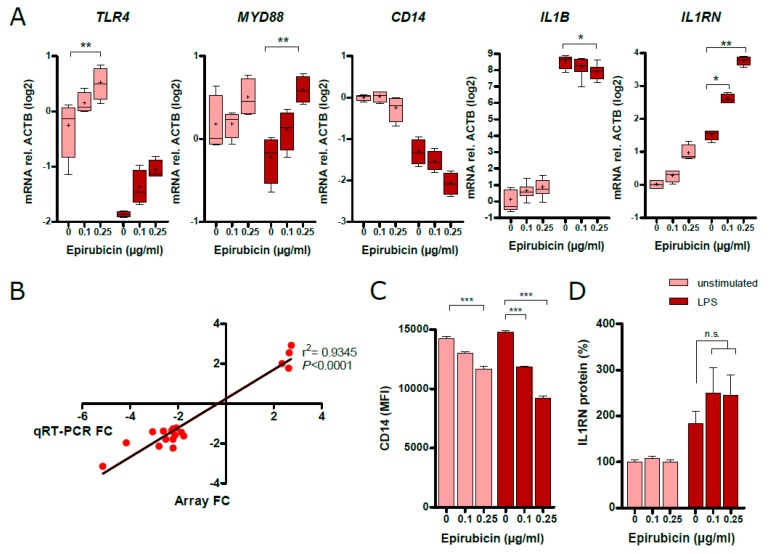
Validation of microarray data. (**A**) Quantitative real-time PCR (q-RT-PCR)-based gene expression analysis of *TLR4*, *MYD88*, *CD14*, *IL1B*, and *IL1RN* of PMA-activated, epirubicin-treated THP-1 in the presence or absence of LPS (100 ng/mL, 3 h). Results were measured in duplicates in 4 independent experiments, and were normalized to ACTB and depicted in log2 scale. (**B**) Correlation of fold changes obtained from microarray (Array FC) with fold changes obtained from qRT-PCR of 18 genes from LPS core set genes. FC: fold change. (**C**) FACS analysis in mean fluorescence intensity (MFI), indicating the surface expression of CD14 on PMA-activated, epirubicin-treated THP-1 in the presence or absence of LPS (100 ng/mL, 3 h). Results were measured in duplicates in 3 independent experiments, shown as means ± SEM. (**D**) Release of IL1RN protein (IL-1RA) from PMA-activated, epirubicin-treated THP-1 in the presence or absence of LPS (100 ng/mL, 3 h). Results shown represent means ± SEM from 3 independent assays. Statistical tests: (**A**,**C**,**D**) one-way ANOVA with Bonferroni correction, (**B**) linear regression analysis; * *p* < 0.05, ** *p* < 0.01, *** *p* < 0.001.

TLR4-related genes, such as *CD14*, *TLR4*, *MYD88*, *IL1B*, and *IL1RN*, which encode the antagonist of the IL-1 receptor, were differentially regulated in microarray analysis. Validation by qRT-PCR confirmed increased expression of *TLR4*, *MYD88*, and *IL1RN* in response to epirubicin and reduced expression of *IL1B* (Figure 6A). Additionally, we validated the expression of 18 LPS core set genes by qRT-PCR and found that all genes exhibited the same regulatory tendency as found by microarray analysis (Supplementary Figure S7). Moreover, the data obtained from qRT-PCR and microarray was significantly correlated with r^2^ = 0.9345 in linear regression analysis (Figure 6B). Analysis of CD14 surface expression of epirubicin-treated THP-1 revealed a decreased expression with epirubicin treatment in the absence and presence of LPS (Figure 6C). Analysis of cell culture supernatants for IL1RN protein revealed a non-significant trend towards an increased release of this potent anti-inflammatory molecule in the presence of epirubicin (Figure 6D).

### 3.7. Increased ERK Signaling but No Changes in NFκB Activation in Response to Low-Dose Epirubicin

**Figure 7:**
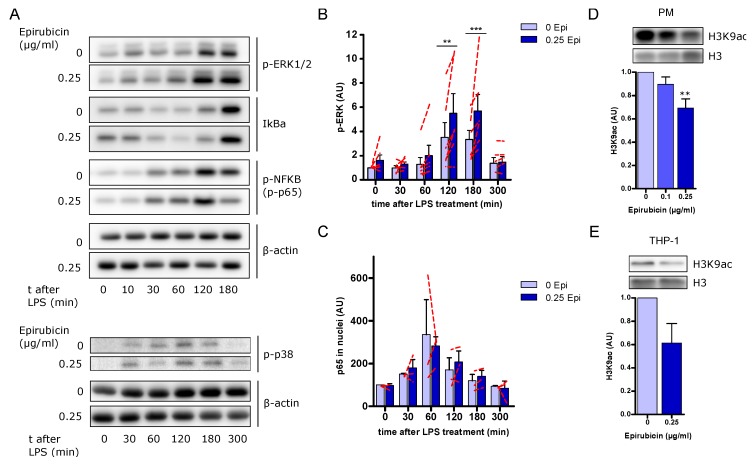
Effects of low-dose epirubicinon cell signaling and histone modifications. (**A**) Representative immunoblots for p-ERK1/2, p-p38, IκBa, and p-p65 in whole cell lysates of PMA-primed, epirubicin-treated THP-1 minutes after LPS treatment. Here, β-actin was used as a loading control. (**B**) Quantitative analysis of p-ERK1/2 protein bands by densitometry using ImageJ software from the whole cell lysate western blots (*n* = 6). Arbitrary units (AU) normalized on the expression of the β-actin. Dashed red lines indicate the results from each experiment. Data shown as mean ± SEM. (**C**) Quantitative analysis of p65 protein bands by densitometry using ImageJ software from nuclei protein western blots (*n* = 3). Arbitrary units (AU) normalized on the expression of the H3 in nuclei. Dashed red lines indicate the results from each experiment. Data shown as mean ± SEM. (**D**,**E**) Representative immunoblot of H3K9ac and quantitative analysis of PM (**D**) or THP-1 (**E**) in response to epirubicin (*n* = 3 each). Immunodetection of H3 was used as loading control. Statistical analysis: two-way ANOVA (**B**), one-way ANOVA (**D**), or paired *t*-test (**E**); ** *p* < 0.01, *** *p* < 0.001.

Several studies have demonstrated the involvement of mitogen-activated protein kinases (MAPK) and NFκB in anthracycline-induced inflammation affecting inflammation, proliferation, growth, and survival [28,29,30,31]. Thus, we investigated the changes in phosphorylation of ERK1/2 (p-ERK1/2) and p38 (p-p38) in order to determine whether the anti-inflammatory properties of low-dose epirubicin are mediated through the regulation of MAPK phosphorylation and regulation of NFκB. As expected, LPS induced the phosphorylation of p65 at position S536 30 min after LPS treatment alongside the degradation of IκBa in PMA-primed THP-1; however, there were no significant differences in p-p65, IκBa, and p-p38 between cells treated and not treated with 0.25 µg/mL epirubicin for 24 h prior to and during TLR4 stimulation (Figure 7A and Supplementary Figure S8). We observed significantly increased phosphorylation of ERK-1/2 in whole cell lysates 2 and 3 h after LPS treatment in epirubicin-treated cells compared to untreated cells (Figure 7B). However, no differences in the nuclear translocation of p65 were detected between both conditions (Figure 7C and Supplementary Figure S9).

### 3.8. Low-Dose Epirubicin Modifies Histone 3 Acetylation on Lysine 9

Further investigating a global suppression of LPS core gene expression alongside the regulation of histone modifying enzymes in microarray analysis, we went on to examine histone modifications at the protein level. Different histone modifications can either activate or suppress adjacent genes by recruiting regulatory proteins [32]. In general, while histone acetylation is associated with the activation of genes, methylation of histones is associated with gene suppression or gene activation, depending on the amino acid position within the histone and the amount of methyl groups added [32]. Whereas the histone-modifications H3K4me3, H3K9me3, H3K27me3, and H3K27ac were not regulated in THP-1 by epirubicin, treatment with epirubicin reduced histone 3 lysine 9 acetylation (H3K9ac) in PM (Figure 7D) and THP-1 (Figure 7E).

## 4. Discussion

Here, we report that epirubicin administered at non-cytotoxic low doses downregulates NLRP3 inflammasome components and reduces the release of cleaved caspase-1 and bioactive IL-1β following NLRP3 activation in a dose-dependent fashion in human macrophages. In addition, epirubicin attenuates inflammatory responses following stimulation with LPS from *Escherichia coli* (TLR4 agonist), with heat-killed *Listeria monocytogenes* (TLR2 agonist), and with the synthetic triacylated lipopeptide Pam3CSK4 (TLR1/2 agonist) in the absence of significant cell death. Given the broad effects on inflammatory pathways observed, we investigated whether low-dose epirubicin exerts its effects via autophagy, DNA damage responses, or cellular signaling pathways, potentially affecting macrophage transcriptional regulation.

The inhibition of autophagy could not restore cytokine release from macrophages at a low dose (0.1 µg/mL), excluding the induction of autophagy [23,33] as a major underlying factor. Only at higher doses (0.5 µg/mL), which induced significant apoptosis and cytotoxicity, did epirubicin induce LC3b protein in the absence or presence of autophagy inhibitors, confirming results of previous studies [23]. This is also in line with our observation that epirubicin at effective doses of 0.1 to 0.25 µg/mL did not result in increased nuclear 53bp1 and γH2AX colocalization, given that DNA damage responses are well-known inducers of autophagy [34]. In addition, our study provides no evidence for a role of altered NFκB expression on gene or protein levels or any disruption of the nuclear translocation of the p65 subunit, even though the TLR4 coreceptor CD14 was downregulated following epirubicin treatment.

Transcriptome analysis revealed a global transcriptional suppression of LPS-dependent genes in response to epirubicin. Epirubicin intercalates into DNA, and was found to inhibit the binding of transcription factors to DNA [27]. Although epirubicin binds to G-C sequences with higher affinity [27], it is not known whether epirubicin targets specific DNA sequences. In our study, epirubicin ameliorated the regulation of a set of genes that are induced in response to LPS and partly depend on NFκB. This result supports the notion that epirubicin either directly inhibits DNA binding of NFκB or other TLR-dependent transcription factors, such as AP1, or reduces DNA enhancer activity in macrophages, finally leading to reduced expression of pro-inflammatory cytokines and regulators. In terms of IL-1β reduction, we found a two-step regulatory mechanism, where pro-IL-1β is transcriptionally silenced first (substrate reduction). Then, the active converting complex (inflammasome, cleaved caspase-1) is reduced via the transcriptional silencing of NLRP3 (enzyme reduction), eventually leading to reduced levels of bioactive IL-1β.

Histone modifications can either activate or suppress adjacent genes by recruiting regulatory proteins [32]. Consistent with the global transcriptional suppression of LPS-dependent genes, we observed a reduced acetylation of H3K9 in response to epirubicin in both PM and THP-1, which can be interpreted as reduced transcriptional activity given that H3K9 is associated with gene activation. This result is consistent with a previous report [27], and yet our study is the first showing H3K9ac reduction at non-cytotoxic low doses of epirubicin in primary macrophages as a possible mechanism to modulate inflammatory responses, including NLRP3 inflammasome activation. However, the transcriptional mediators of epirubicin-mediated macrophage reprogramming are still elusive and need to be identified in further studies.

This study established a new field of application for the licensed anti-tumor drug epirubicin as an anti-inflammatory agent, reducing IL-1β and TNF-α release from macrophages when used at low-doses. Given the increasingly recognized role of NLRP3 for the development of liver injury and fibrosis [6,8,10,11], therapeutic interventions focusing on the epigenetic programming of immune cells and the organ-specific inhibition of inflammasomes represent novel anti-inflammatory strategies, which need to be further investigated in the context of fibrogenesis. Furthermore, the anti-inflammatory properties of epirubicin may be an adverse effect in cancer therapy.

## Supplementary Materials

The following are available online at https://www.mdpi.com/2073-4409/9/1/79/s1. Figure S1: Low-dose epirubicin suppresses the release of bioactive IL-1β. Figure S2: Low-dose epirubicin does not affect LPS/ATP-mediated pyroptosis. Figure S3: Low-dose epirubicin does not affect LPS/ATP-mediated pyroptosis in presence of autophagy inhibitors. Figure S4: Interindividual variability of TNF-α and IL-1β release from primary macrophages. Figure S5: Gating strategy and representative plots of intracellular TNF-staining of PMA-activated THP-1 after LPS treatment. Figure S6: The frequency of TNF-producing LPS-treated PMA-activated THP-1 does not change after incubation with epirubicin. Figure S7: Validation of 18 LPS core set genes from microarray. Figure S8: Effects of low-dose epirubicin on phospho-p38 (Thr180/Tyr182) and phospho-NF-kB-p65 (Ser536) in THP1 cells. Figure S9: Representative immunoblot of the p65 subunit of NFkB in the nuclear fraction of THP-1 cells in absence or presence of epirubicin at 0.25 µg/mL.; Table S1: List of primers used in qRT-PCR. Table S2: Array all data. Table S3: GSEA Pathways in steady state. Table S4: LPS core genes.
