# Supplementary material for: Transcriptional Suppression of the NLRP3 Inflammasome and Cytokine Release in Primary Macrophages by Low-Dose Anthracyclines
Source: Cells. 2019 Dec 28;9(1):79. doi: 10.3390/cells9010079 (PMC7016608; doi:10.3390/cells9010079)
Supplement: Supplementary file 1 [file cells-09-00079-s001.zip › cells-651528-supplementary-final/cells-651528-supplementary-final.pdf]

cells

MDPI

Supplementary Materials for

Transcriptional Suppression of the NLRP3 Inflammasome and Cytokine Release in Primary Macrophages by Low-Dose Anthracyclines

Nilay Köse-Vogel [1], Sven Stengel [1], Elena Gardey [1], Tatiana Kirchberger-Tolstik [1], Philipp A. Reuken [1], Andreas Stallmach [1] and Tony Bruns [1,2,3,*]

[1] Department of Internal Medicine IV (Gastroenterology, Hepatology, and Infectious Diseases), Jena University Hospital, Friedrich Schiller University of Jena, 07747 Jena, Germany; nilay.koese@med.uni-jena.de (N.K.-V.); sven.stengel@med.uni-jena.de (S.S.); elena.gardey@med.uni-jena.de (E.G.); tatiana.kirchberger-tolstik@med.uni-jena.de (T.K.-T.); philipp.reuken@med.uni-jena.de (P.A.R.); andreas.stallmach@med.uni-jena.de (A.S.)

[2] The Center for Sepsis Control and Care (CSCC), Jena University Hospital, Friedrich Schiller University of Jena, 07747 Jena, Germany

[3] Department of Internal Medicine III, University Hospital RWTH Aachen, 52074 Aachen, Germany

* Correspondence: tbruns@ukaachen.de

Supplementary Materials: The following are available online at www.mdpi.com/xxx/s1, Figure S1: Low-dose epirubicin suppresses the release of bioactive IL-1β, Figure S2: Low-dose epirubicin does not affect LPS/ATP-mediated pyroptosis. Figure S3: Low-dose epirubicin does not affect LPS/ATP-mediated pyroptosis in presence of autophagy inhibitors. Figure S4: Interindividual variability of TNF-α and IL-1β release from primary macrophages. Figure S5: Gating strategy and representative plots of intracellular TNF-staining of PMA-activated THP-1 after LPS treatment. Figure S6: The frequency of TNF-producing LPS-treated PMA-activated THP-1 does not change after incubation with epirubicin. Figure S7: Validation of 18 LPS core set genes from microarray. Figure S8: Effects of low-dose epirubicin on Phospho-p38 (Thr180/Tyr182) and Phospho-NF-kB-p65 (Ser536) in THP1 cells. Figure S9: Representative immunoblot of the p65 subunit of NFkB in the nuclear fraction of THP-1 cells in absence or presence of epirubicin at 0.25 µg/ml. Table S1: List of primers used in qRT-PCR, S2: Array all data, Table S3: GSEA Pathways in Steady state, Table S4: LPS Core Genes

This file includes: Supplementary Figures S1 to S9 and Table S1

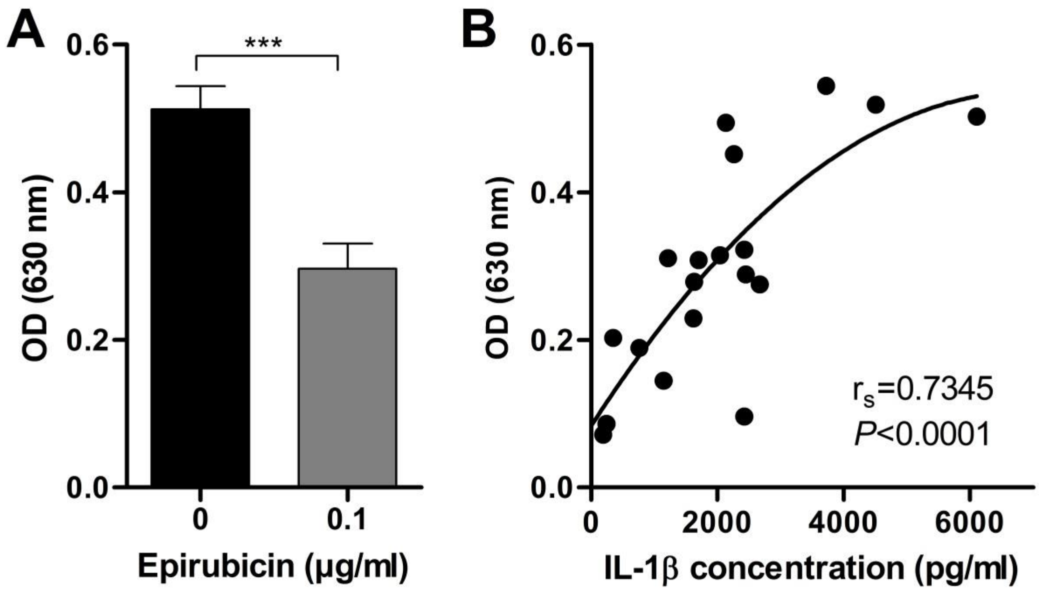

Figure S1: Low-dose epirubicin suppresses the release of bioactive IL-1β. (A) Release of mature IL-1β from peritoneal macrophages (PM) PMs as OD values from HEK-Blue IL-1β reporter assay. (B) Correlation analysis of IL-1β concentration of 0.1 µg/ml epirubicin pretreated cells, from ELISA and OD values from HEK-Blue IL-1β reporter assay. Results shown represent 19 independent experiments (Cells from 19 different patients). Statistical analysis: Wilcoxon signed rank test (A) Spearman’s rank correlation (B). *** P<0.001

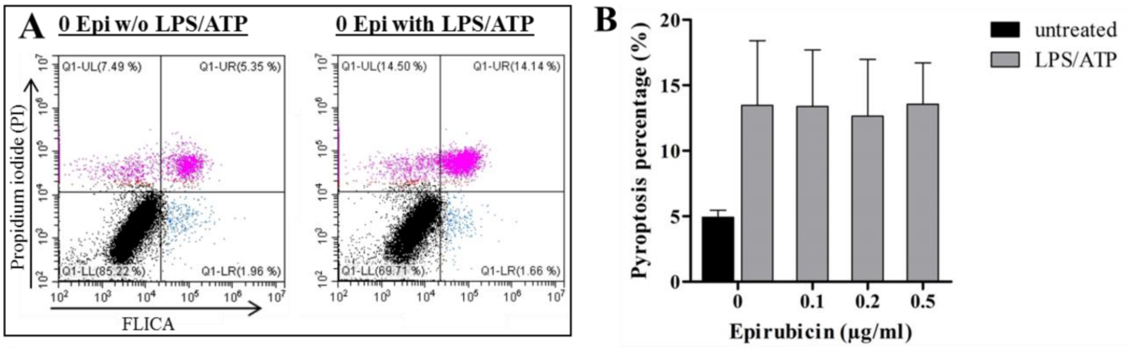

Figure S2: Low-dose epirubicin does not affect LPS/ATP-mediated pyroptosis. (A) Representative flow cytometry of FLICA/PI-stained primary peritoneal macrophages in absence or presence of NLRP3 activation with LPS/ATP. (B) Pyroptotic cells (FLICA+PI+) were quantified after incubation with increasing concentrations of epirubicin (Epi). Results represent means ± SEM from 4 independent patients.

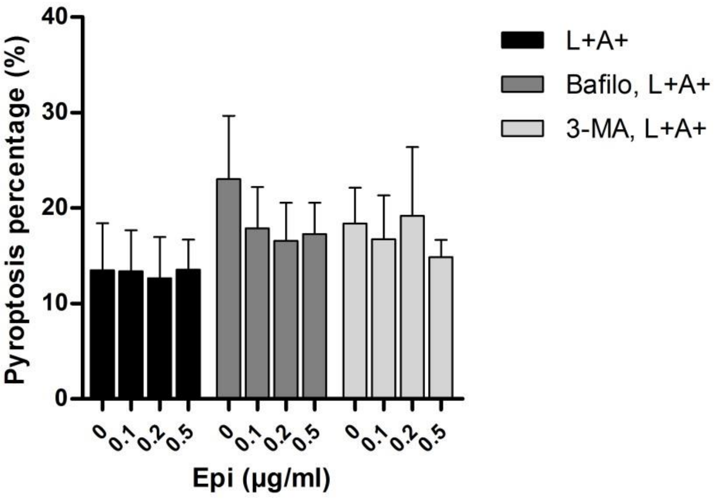

Figure S3: Low-dose epirubicin does not affect LPS/ATP-mediated pyroptosis in presence of autophagy inhibitors. Autophagy was inhibited by 300 nM bafilomycin and 10 mM 3-MA for 4 h. Pyroptosis was determined by flow cytometry (FLICA+PI+). Results represent means ± SEM from 4 independent experiments.

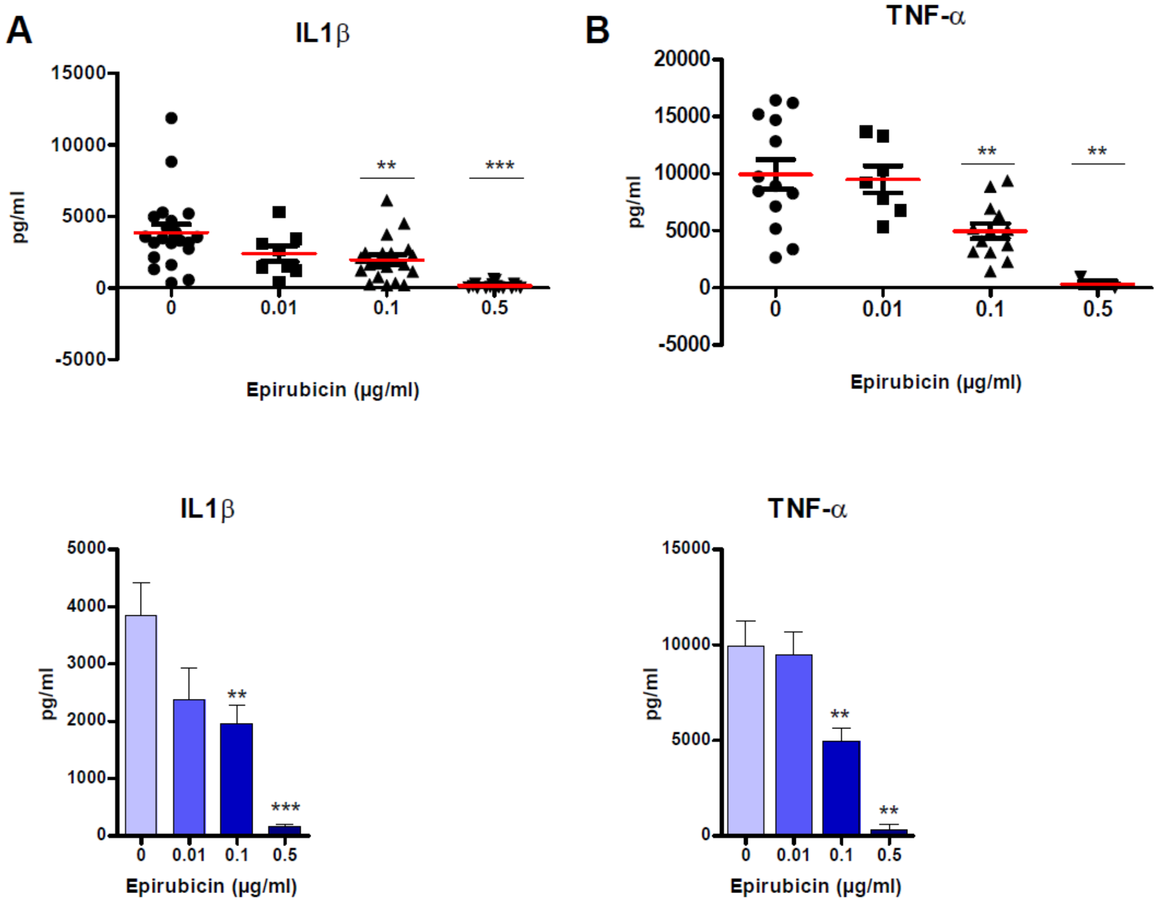

Figure S4: Interindividual variability of TNF-α and IL-1β release from primary macrophages. (A) IL-1β and (B) TNF-α release from primary peritoneal macrophages into cell culture supernatant. PM were incubated with increasing doses of epirubicin for 24 h and subsequently stimulated with LPS/ATP. Supernatants were analyzed by ELISA. Data are given as scatter plots (top panels) and bar charts (bottom) indicating means with S.E.M.. Statistical analysis: One-way ANOVA with Tukey's multiple comparison test. **$P<0.01$, *** $P<0.001$

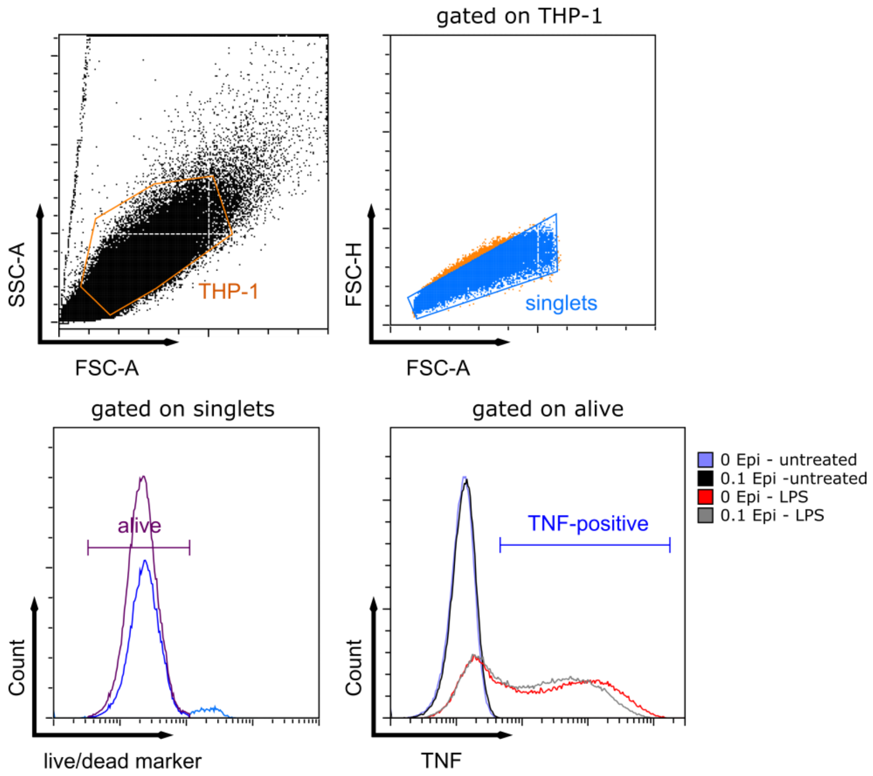

Figure S5: Gating strategy and representative plots of intracellular TNF-staining of PMA-activated THP-1 after LPS treatment

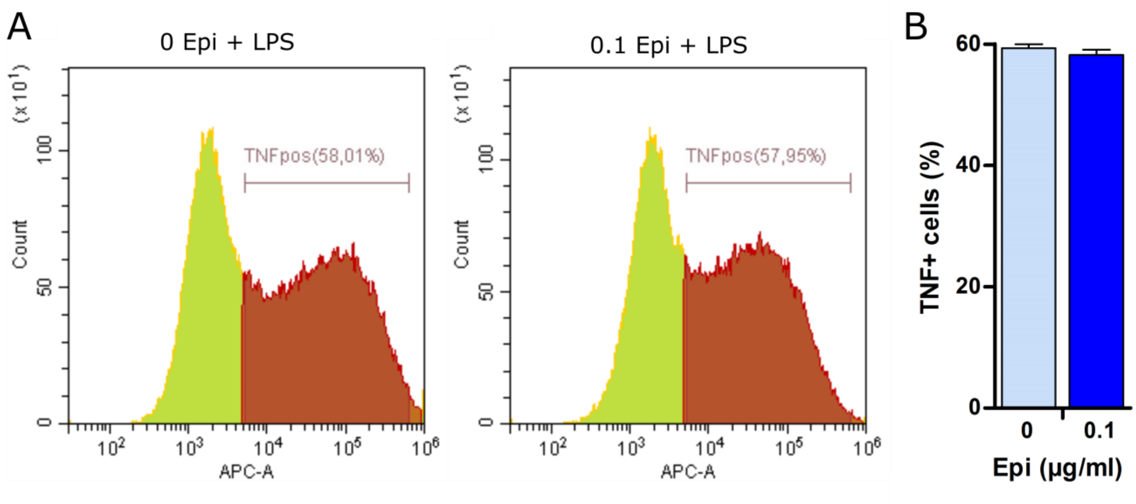

Figure S6: The frequency of TNF-producing LPS-treated PMA-activated THP-1 does not change after incubation with epirubicin. (A) Representative flow cytometry histograms of TNF positive (TNFpos) cells in absence or presence of treatment with epirubicin at 0.1 µg/ml. (B) Percentage of TNF+ cells determined after intracellular staining of TNF-alpha. Results shown represent means ± SEM from 3 independent assays.

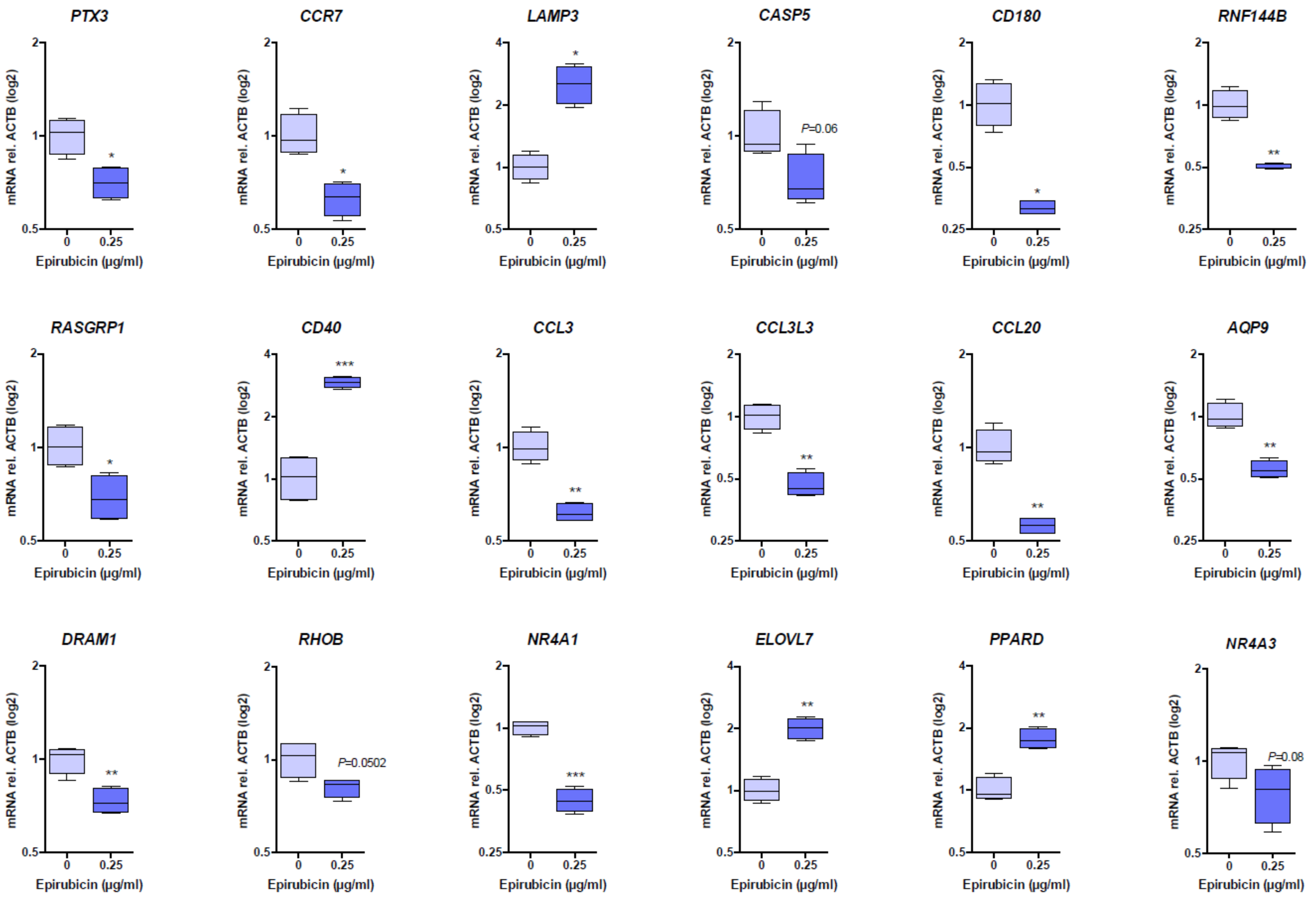
PTX3
CCR7
LAMP3
CASP5
CD180
RNF144B
RASGRP1
CD40
CCL3
CCL3L3
CCL20
AQP9
DRAM1
RHOB
NR4A1
ELOVL7
PPARD
NR4A3
P=0.06
P=0.0502
P=0.08
mRNA rel. ACTB (log2)
Epirubicin (µg/ml)
0
0.25

Figure S7: Validation of 18 LPS core set genes from microarray. Quantitative real time PCR (q-RT-PCR) based gene expression analysis of 18 genes from LPS core set PMA-activated, epirubicin treated THP-1 in the presence of LPS (100 ng/ml, 3 h). Results were measured in duplicates in 4 independent experiments, and were normalized to ACTB, and depicted in log2 scale. Statistical test: t-test with Welch correction. * $P<0.05$, ** $P<0.01$, *** $P<0.001$

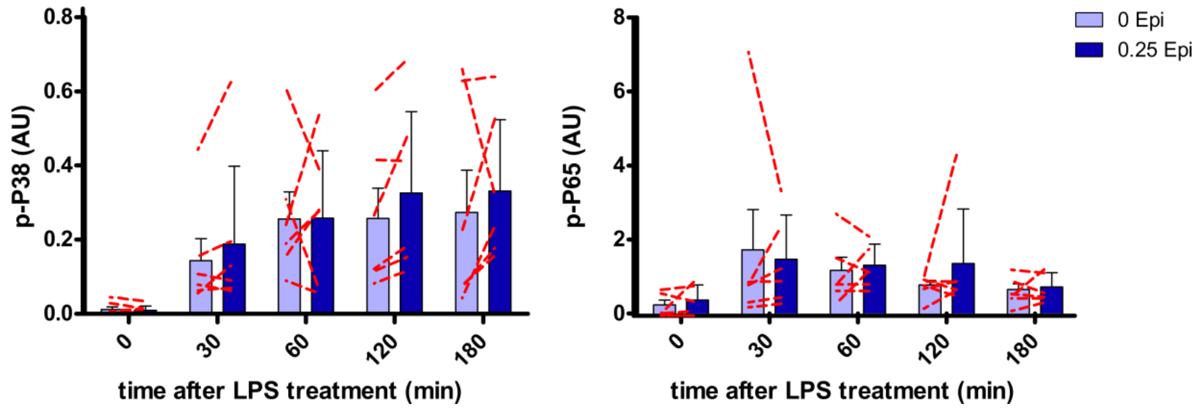

Figure S8: Effects of low-dose epirubicin on Phospho-p38 (Thr180/Tyr182) and Phospho-NF-kB-p65 (Ser536). Total Protein from cell lysates (n=6) were analysed for the expression of Phospho-p38 or Phospho-NF-kB-p65 by Western blot. Quantitative analysis of specific protein bands was performed by densitometry using ImageJ software and target protein amounts were normalized against β-Actin expression. Arbitrary units (AU) of normalized target protein amounts. Data shown in mean ± SEM**.**

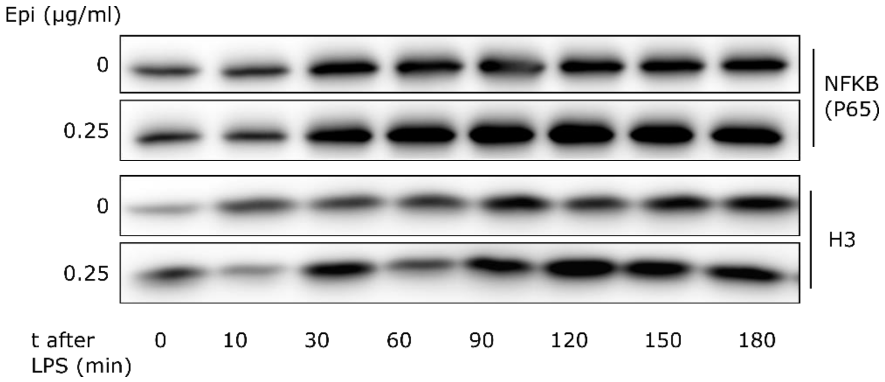

Figure S9: Representative immunoblot of the p65 subunit of NFkB in the nuclear fraction of THP-1 cells in absence or presence of epirubicin at 0.25 µg/ml. Cells were subsequently treated with 100 ng/ml LPS. H3 served as a loading control.

Table S1: List of primers used in qRT-PCR

**Gene**	**Nucleotide sequence in 5′ to 3′ direction**
ACTB	F: CATGTACGTTGCTATCCAGGC<br>R: CTCCTTAATGTCACGCACGAT
AQP9	F: ACTGCTGATCGTGGGAGAAAA<br>R: GCGTTCGCCAGAGATAGATACG
CASP5	F: GGTGAAAAACATGGGGAACTC<br>R: TGAAGAACAGAAAGCAATGAAGT
CCL20	F: TGCTGTACCAAGAGTTTGCTC<br>R: CGCACACAGACAACTTTTTCTTT
CCL3	F: GCAACCAGTTCTCTGCATCA<br>R: TGGCTGCTCGTCTCAAAGTA
CCL3L3	F: TCTGCAACCAGGTCCTCTCT<br>R: TTTCTGGACCCACTCCTCAC
CCR7	F: TGAGGTCACGGACGATTACAT<br>R: GTAGGCCCACGAAACAAATGAT
CD14	F: AGAGGTTCGGAAGACTTATCG<br>R: TCGGAGAAGTTGCAGACGC
CD180	F: ACTTCCCAGCACGGAATCTG<br>R: GTTCAGGCTTAGGTTGATGGC
CD40	F: GGCCAAGAAGCCAACCAATAA<br>R: GAAGATCGTCGGGAAAATTGAT
DRAM1	F: TGTCTGTGCTTCACTAATTTCCA<br>R: TCACAGATCGCACTCACTACG
ELOVL7	F: GGCCAGCCTACCAGAAGTAT<br>R: ATGATGCACGCAAAGACTGG
IL1RN	F: AAGATGTGCCTGTCCTGTGT<br>R: CGCTTGTCCTGCTTTCTGTT
LAMP3	F: TGAAAACAACCGATGTCCAA<br>R: TCAGACGAGCACTCATCCAC
MYD88	F: GGATGGTGGTGGTTGTCTCT<br>R: TGCCTTGTACTTGATGGGGA
NR4A1	F: CACATTGTTGCCAAGACCTG<br>R: TGCTGGTGTCCCATATTGG
NR4A3	F: GAATCAGCCTTTTTGGAGCTGT<br>R: CATTGCAGAACACAAACTTATCTTCA

PPARD	F: GTCACACAACGCTATCCGTTT<br>R: AGGCATTGTAGATGTGCTTGG
PTX3	F: TCTCTGGTCTGCAGTGTTGG<br>R: TGAAGAGCTTGTCCCATTCC
RASGRP1	F: GGCTCAAGGAGACAAGTTCG<br>R: GAAGTCGGTGCACTCTCCATA
RHOB	F: CGGACTCGCTGGAGAACA<br>R: GAGGTAGTCGTAGGCTTGGAT
RNF144B	F: CTGGTAGGCTCCACTATCTCG<br>R: GGGCAAGTGATGGGAGACC
TLR4	F: ACCTCCCCTTCTCAACCAAG<br>R: GGCTCTGATATGCCCCATCT
